# Are patients on oral anticoagulation therapy aware of its effects? A cross-sectional study from Karachi, Pakistan

**DOI:** 10.1186/s13104-020-05119-w

**Published:** 2020-06-09

**Authors:** Ibrahim Zahid, Syed Wajih Ul Hassan, Nida Sehar Bhurya, Sheena Nadeem Alam, Choudhary Ahmed Hasan, Bakht Hussain Shah, Fasiha Bakhtawar Fatima, Ayesha Ahmed, Syed Sabih Ul Hassan, Javeria Hayat, Aqsa Zulfiqar, Rija Sheikh, Momin Aziz, Rabbia Siddiqi, Kaneez Fatima, Muhammad Shahzeb Khan

**Affiliations:** 1 Dow Medical College, Dow University of Health Sciences, Karachi, Pakistan; 2 Dr. Ruth K.M. Pfau Civil Hospital, Karachi, Pakistan; 3 John H Stroger Jr. Hospital of Cook County, Chicago, IL USA

**Keywords:** Anticoagulants, Warfarin, International normalised ratio

## Abstract

**Objective:**

Oral anticoagulants are one of the most frequently used medications. However, these drugs have a range of side effects including potential life-threatening complications. Little is known regarding the awareness of its side effect profile amongst the patients in Pakistan. Therefore, the aim of this study was to assess the knowledge of oral anticoagulant therapy and its side effects among its users.

**Results:**

The mean age was 48.9 ± 15.2 years. Median scores of the participants for knowledge regarding oral anticoagulants and warfarin were 48.7 (8.3–91.7) and 10.3 (0.0–70.0) respectively. Of 207 patients, most notably, 65.7% did not know what side effects to be wary of or how to reduce their occurrence; and most patients were unaware of the interaction between oral anticoagulant drugs and over-the-counter substances such as aspirin, herbal medicines and alcohol. Knowledge of international normalised ratio (INR) was extremely poor with more than 75% of the population not being aware of the target INR range during warfarin therapy. Higher level of education was significantly associated with better knowledge scores. Overall, knowledge of oral anticoagulant therapy and INR monitoring is extremely poor among oral anticoagulant users.

## Introduction

With several indications ranging from atrial fibrillation to mechanical heart valves, oral anticoagulants (OAC) use is quite pervasive in modern society [1, 2]. Drugs like warfarin and apixaban impede clotting of blood and hence are used where there is a high risk of thrombosis. However, OAC especially warfarin, have a narrow therapeutic index which requires careful dosing and monitoring to avoid both ineffectual doses and side effects [3]. Side effects include, but are not limited to, bleeding diatheses, thromboembolism, and hypersensitivity reactions [4].

Despite the prevalence of their use and risk of serious adverse effects, several studies have shown that most patients do not have adequate knowledge of the OAC they are using [5, 6]. Other studies have found that long-term outcomes of OAC are better when patients possess greater knowledge regarding OAC medications [7, 8]. The improved outcome has been attributed to several factors such as increased adherence to dosing schedules, regulation of diet to prevent interactions with the anticoagulants, and closer monitoring of the side effects of anticoagulation therapy. Meanwhile, awareness of Internal Normalised Ratio (INR) monitoring is equally important for improved outcomes. Previous studies have shown a positive correlation between the knowledge of patients’ warfarin therapy and the INR values lying within the target ranges [8, 9]. Poor treatment outcomes were seen in patients with lack of medication adherence and knowledge deficits [9]. Data particularly regarding knowledge of OAC is highly insufficient in Pakistan; upon assessing the frequency of achieving target INR during warfarin therapy, a study from 2012 revealed poor monitoring and control without precisely identifying the reason behind it [10]. Considering the scarcity of relevant data in Pakistan, our study specifically aims to assess the level of knowledge patients have about their OAC.

## Main text

### Methods

This quantitative, multi-centric, cross-sectional study was conducted from January to June 2019 at Civil Hospital Karachi and National Institute of Cardiovascular Diseases, Karachi Pakistan. Ethical approval was obtained from Institutional Review Board of Dow University of Health Sciences, Pakistan.

Patients taking any OAC drugs for at least 1 month were included in the study through non-probability convenience sampling except paediatric patients, medical staff, nurses, medical students, healthcare professionals and those unwilling to participate. A written informed consent was taken from the patients, stating the aim of the study and its impact; confidentiality of patients was ensured. Based on the assumption that 74.1% had adequate knowledge regarding OAC [11], and taking a 5% margin of error and 90% confidence level, the calculated sample size was 207. After adjusting for 10% non-response (or partially filled forms), 230 patients were recruited for the study.

Face-to-face interviews were conducted with the patients using the Oral Anticoagulation Knowledge Tool (AKT) which was shown to have acceptable validity and reliability in a previous study [12]. The questionnaire is divided into three parts (see Additional file 1: Appendix S1). The first section is related to the demographic characteristics of patients. The second part consisted of 20 questions to assess the patients knowledge about OAC (section 2), and the final 8 questions (section 3) were exclusive to patients on warfarin therapy.

For each question, ‘1’ mark was awarded for each correct answer and ‘0’ for each wrong answer, except for questions ‘18’ and ‘19’ in ‘section 2’ and ‘6b’ in ‘section 3’. In these questions 1 mark was given for each correct point out of 3. Section 2 was scored out of 24, and section B, which was only filled by patients on warfarin therapy, was scored out of 10. Final scores were presented as a percentage of correct answers for all the participants in the study. A cut off of > 50% was considered as an adequate knowledge score.

Data were analyzed using IBM Statistical Package for the Social Sciences (SPSS) version 22. Knowledge of OAC was evaluated through total oral AKT scores by simply counting the number of correct answers and calculating their percentages. Frequencies and percentages were calculated for all categorical variables. Overall mean and median scores were calculated for both sections, and mean scores of both sections were also reported for all demographic groups. Mann–Whitney U Test (for 2 groups) and Kruskal–Wallis Test (for more than 2 groups) were applied to compare mean scores among different demographic characteristics. *p *< 0.1 was considered to be significant.

### Results

The study had a response rate of 90%. As presented in Table 1, the mean age was 48.9 ± 15.2. Half of the study population was male (n = 105; 50.7%). Most of the participants (n = 138; 66.7%) had no formal education and almost half (n = 98; 47.3%) had a monthly family income of $100–200.

**Table 1:** Demographics of study population and mean scores

Demographics	Frequency n (%)	Mean percentage score ± SD			
		Knowledge of anticoagulant	P-value	Knowledge specific to warfarin	P-value
Age groups (years)			0.452		0.277
Mean ± SD (years)	48.9 ± 15.2				
≤ 30	27 (13.0)	46.5 ± 16.3		16.5 ± 13.4	
31–50	92 (44.4)	50.1 ± 16.9		13.2 ± 16.9	
> 50	88 (42.5)	50.6 ± 15.5		15.4 ± 16.9	
Gender			0.055*		0.185
Male	105 (50.1)	51.9 ± 16.8		13.3 ± 16.8	
Female	102 (49.3)	47.7 ± 15.4		15.7 ± 16.0	
Marital status			0.666		0.366
Single	38 (18.4)	48.2 ± 15.1		15.8 ± 14.8	
Married	169 (81.6)	50.2 ± 16.4		14.2 ± 16.8	
Highest level of education			< 0.001*		0.977
High school or equivalent	56 (27.1)	56.7 ± 16.3		16.2 ± 20.4	
Technical or vocational education	5 (2.4)	56.7 ± 11.3		12.5 ± 9.6	
Bachelor’s and above	8 (3.9)	60.4 ± 19.9		18.3 ± 24.8	
No formal education	138 (66.7)	46.2 ± 15.0		13.6 ± 13.6	
Occupation			0.228		0.434
Service and sales worker	9 (4.4)	53.2 ± 16.8		11.7 ± 16.0	
Skilled agricultural, forestry and fishery workers	6 (2.9)	38.9 ± 5.0		12.5 ± 18.9	
Craft and related trades workers	12 (5.8)	54.2 ± 15.2		12.9 ± 14.9	
Plant and machine operators and assemblers	10 (4.8)	54.2 ± 13.6		14.3 ± 19.0	
Elementary occupations	33 (15.9)	52.3 ± 16.3		9.6 ± 16.5	
Housewife	84 (40.6)	47.6 ± 15.7		15.9 ± 16.7	
Unemployed/retired	40 (19.3)	48.6 ± 16.0		15.9 ± 13.4	
Managers/professionals/technicians and associate professionals	13 (6.3)	57.4 ± 21.7		18.3 ± 21.2	
Family income (USD)			0.182		0.032*
< 100	80 (38.6)	48.9 ± 17.6		17.5 ± 15.6	
100–200	98 (47.3)	48.5 ± 13.7		13.4 ± 15.6	
200–500	26 (12.6)	56.6 ± 18.1		10.5 ± 19.4	
> 500	3 (1.5)	61.1 ± 26.7		20.0 ± 26.5	

*Significant at 10% level

When assessed for knowledge, most of the participants were using warfarin (n = 147; 71%), and about one-quarter were taking rivaroxaban (n = 45; 21.7%). Most patients (n = 116; 56.1%) knew that these drugs actually prevent blood from clotting but more than a quarter of the participants did not know the drug mechanism (n = 66; 31.9%). More than half the patients said it is important to take medicine same time each day (n = 139; 67.1%) and 64.7% (n = 134) disagreed to double the dose if missed. Nearly half of the study population believed that missing a dose could worsen their condition (n = 87; 42%). Majority of patients believed that they should continue with the drug even if they felt better (n = 111; 53.6%). Majority was unsure if it is safe to take anti-inflammatory drugs or vitamins, herbal medicines or alcohol with their OAC.

There was a positive response from the patients regarding informing their physician or dentist about their OAC (n = 161; 77.8%). Most patients were unaware about main side effects of their anticoagulant or over all three side effects to watch out for (n = 113; 54.6%). When patients were asked about the best step if they consume too much of this medicine, most of them opted for consulting their doctor (n = 110; 53.2%). This is displayed in Table 2.

**Table 2:** Knowledge of OAC

Knowledge questions	Frequency n (%)
Name of OAC	
Warfarin	147 (71)
Rivaroxaban	45 (21.7)
Don’t know the name	15 (7.2)
Why has your doctor prescribed this medicine	
Arrhythmias	14 (6.8)
Blood thinning	47 (22.7)
Cardiac issue/chest pain	20 (9.7)
DVT	15 (7.2)
MI	39 (18.8)
Prosthetic valve	26 (12.6)
Don’t know	26 (12.6)
Others	20 (9.7)
How does this medicine work in your body	
Lowers BP	16 (7.7)
Prevents blood from clotting	116 (56.1)
Lowers heart rate	9 (4.3)
Don’t know	66 (31.9)
How many times a day do you need to take this medicine	
Once	171 (82.6)
Twice	22 (10.6)
Thrice	4 (1.9)
Don’t know	10 (4.8)
For how long do you need to take this medicine	
3 months	15 (7.2)
6 months	18 (8.7)
1 year	19 (9.2)
Lifelong	89 (43)
Don’t know	66 (31.9)
Why is it important to take this medicine exactly as the doctor has told you	
Too much of this can cause bleeding	44 (21.3)
Skipping a dose can cause bleeding	2 (1)
It interacts with food, so changing the dose/timing can be hazardous	6 (2.9)
Don’t know	155 (74.9)
Is it important to take this medicine at the same time each day	
Yes	139 (67.1)
No	31 (15)
Not sure	37 (17.9)
Is it okay to double the next dose of this medicine if you missed a dose	
Yes	23 (11.1)
No	134 (64.7)
Not sure	50 (24.2)
Could missing one dose worsen your condition	
Yes	87 (42)
No	68 (32.9)
Not sure	52 (25.1)
Is it appropriate to stop taking this medicine once you feel better	
Yes	46 (22.2)
No	111 (53.6)
Not sure	50 (24.2)
Is it safe to take anti-inflammatory meds while on OAC	
Yes	67 (32.4)
No	41 (19.8)
Not sure	99 (47.8)
Is it safe to take vitamins, herbal meds without consulting doctor	
Yes	66 (31.9)
No	64 (30.9)
Not sure	77 (37.2)
Is it beneficial to take more medicine than prescribed	
Yes	11 (5.3)
No	126 (60.9)
Not sure	70 (33.8)
Will drinking too much alcohol increase the risks of this med	
Yes	70 (33.8)
No	10 (4.8)
Not sure	127 (61.4)
Would you inform surgeon, dentist, doc about your meds	
Yes	161 (77.8)
No	19 (9.2)
Not sure	27 (13)
Is it imp that all healthcare practitioners know about this med	
Yes	161 (77.8)
No	23 (11.1)
Not sure	23 (11.1)
Most important side effect of this medicine	
Bleeding	49 (23.7)
Others	20 (9.7)
Don’t know	136 (65.7)
Correct side effects identified	
All three	56 (27.1)
Two correct	22 (10.6)
One correct	7 (3.4)
None	5 (2.4)
Don’t know	117 (56.5)
Side effect identified	
Bleeding gums	72 (19.7)
Prolonged nosebleeds	50 (13.7)
Severe bruising	32 (8.8)
Blood in urine	64 (17.5)
Insomnia	10 (2.7)
Loss of apetite	20 (5.5)
Don’t know	117 (32.1)
How to reduce side effects	
Monitor INR regularly	15 (4.9)
Monitor	44 (14.4)
Sleeping on time	21 (6.9)
Eating less food	14 (4.6)
Avoid things that could cause cuts/injuries	31 (10.1)
Proper dosing	53 (17.3)
Don’t know	128 (41.8)
Best step if you take too much of this medicine	
Skip the next dose	35 (16.9)
Consult my doctor	110 (53.1)
Be alert for signs of side effects	15 (7.2)
Don’t know	47 (22.7)

*OAC* oral anticoagulants, *INR* international normalized ratio

Table 3 shows knowledge of participants specific to warfarin therapy. More than three-quarters of the study population (n = 116; 78.9%) did not know about the target INR range during warfarin therapy. Most of the patients were unware of their last INR reading (n = 78; 53.1%). Only 16.4% believed that regular INR tests were necessary to know if the medicine is working. More than half of the participants (n = 98; 66.7%) were not sure if diet had any effect on their warfarin therapy, and a fairly small percentage agreed to this idea (n = 24; 16.3%).

**Table 3:** Knowledge specific to Warfarin therapy (n = 147)

	Frequency n (%)
What is your target INR range	
< 1.0	17 (11.6)
1.0 to 1.9	5 (3.4)
2.0–3.0	9 (6.1)
Don’t know	116 (78.9)
Last INR reading	
< 1.0	1 (0.7)
1.0–2.0	49 (33.3)
2.1–3.0	14 (9.5)
> 3.0	5 (3.4)
Don’t know	78 (53.1)
Are regular INR tests necessary to know that the medicine is working	
Yes	34 (23.1)
No	8 (5.4)
Not sure	105 (71.4)
Is an INR above target good for health	
Yes	8 (5.4)
No	20 (13.6)
Not sure	119 (81.0)
Is an INR below target bad for health	
Yes	16 (10.9)
No	10 (6.8)
Not sure	121 (82.3)
Is it possible what you eat to affect your Warfarin therapy	
Yes	24 (16.3)
No	25 (17.0)
Not sure	98 (66.7)
Vitamins that can affect your OAC therapy	
Vitamin A	1 (0.7)
Vitamin B	5 (3.4)
Vitamin K	6 (4.1)
Don’t know	131 (89.1)
Others	4 (2.7)

*INR* international normalized ratio

Additional file 2: Table S1 further elaborates on the mean knowledge scores of the population. Only 41.5% of people had good knowledge and were able to score above 50% mark when assessed for knowledge about their OAC, and less than 2% were above this mark when assessed for knowledge specific to warfarin. Upon assessing knowledge scores according to the demographic characteristics, male gender and having a minimum of bachelors level education was significantly related to higher anticoagulant knowledge scores; monthly income greater than USD 500 was associated with higher knowledge of warfarin, as shown in Table 1.

### Discussion

Similar to researches conducted by Shrestha et al. [5], Hu et al. [13] and Baker et al. [11], our study showed that patients have low knowledge towards their prescribed OAC. Taking the demographics into account, one can reasonably attribute the lack of formal education and a language barrier as the primary causes of this gap in knowledge. However, previous literature regarding the association of knowledge with age has mixed findings. Some researchers conclude that participants of younger age and higher level of education scored better [14, 15], whereas others showed older age to positively influence the scores [16]. Our study does not show any significant relationship of knowledge scores with participants’ age.

Findings of our study indicate that only 27.1% of the participants correctly identified bleeding as the most important side effect of this medication, particularly bleeding from gums, prolonged nosebleeds and blood in urine. The majority of the sample (65.7%) did not know what side effects to be wary of or how to reduce their risk. These findings prove to be an alarming indicator of lack of knowledge as most of these participants had been asked to continue this medicine for life and their lack of knowledge about possible side effects is concerning. This gap in knowledge is common in reference to many studies, one of which reported only 42% of their sample population to be aware of any possible side effects of warfarin therapy [17].

Another particular area of concern was the inadequate patient understanding of warfarin’s interactions. Majority of the participants were unaware of the interaction of OAC drugs with over the counter pills like aspirin, herbal medicines and alcohol. Drugs such as Non-Steroidal Anti-Inflammatory Drugs (NSAIDs) and aspirin inhibit platelet function therefore when used with oral anticoagulants, they may increase the risk of bleeding [18]. There is a high prevalence of multiple NSAIDs prescription in Pakistan [19], an alarming fact when considering that NSAIDs increase the risk of gastrointestinal bleeding when used in combination with warfarin, and that only a minority of our sample was aware of this interaction. Alcohol, on the other hand, interacts with the cytochrome P450 system, responsible for metabolizing warfarin. Only one-third of the study population understood the risk of concurrent use of alcohol and an even smaller percentage to that with NSAIDs. A similar finding was obtained by Shrestha et al. [5], who reported that 94.1% of their sample did not know which drinks could decrease warfarin’s effectiveness. This is particularly worrying when taking into consideration the fact that excess alcohol can increase the risk of major bleeding when taken with warfarin, and only 23.7% of our sample could identify bleeding as the most important side effect of oral anticoagulants. These findings were similar to results obtained by Roche et al. [6], Yahaya et al. [20] and Campbell et al. [21]. A lack of understanding with regards to dietary restrictions was also evident. A majority of the study population did not consider vitamin supplementation or the use of vegetables, such as kale or spinach, deleterious to their warfarin therapy; a finding consistent with a study conducted by Nasser et al. [22].

The sample population barely knew about INR, its target values and the importance of therapeutic INR range; attaining a mean score of only 14.6 (± 16.4)%. Furthermore, a research also found that even though young and educated people are likely to have better knowledge about their anticoagulant medicines, this does not affect their INR control or episodes of bleeding and/or thrombosis [23]. On the contrary, a research in Saudi Arabia showed INR control to be positively influenced by knowledge about their OAC [24]. Maintaining a stable INR is an important indicator of adequate anticoagulation in the body, because supratherapeutic value increases the probability of bleeds and subtherapeutic value may cause thrombosis [25]. Within our sample population, the ability to self-monitor is likely to be reduced due to their lack of adequate knowledge about INR, increasing the probability of patients being under or over treated.

A positive result identified in our patient population indicated that over three quarters of people agreed it was important for their healthcare providers to know they were taking an OAC, and the similar proportion of people said that they would usually inform their doctors about their drug history. In contrast, Khudair et al. [26] found that most participants did not know the importance of informing healthcare professionals if they were on warfarin.

### Recommendations

One way to fill this knowledge gap is to ensure that patients receive proper counselling whenever they are prescribed a new drug, and at regular intervals thereafter. Healthcare providers should allocate more time to counselling their patients, focusing on drug side effects and interactions, as well as the proper course of action to take once adverse effects occur. Pamphlets with information on the prescribed drug should also be provided to patients, giving them a chance to refresh their knowledge at their own convenience. More emphasis should also be placed on the need for INR monitoring after beginning warfarin therapy, as this is one area where patient knowledge is severely lacking. Considering the high risk profile of the drugs, follow ups with the patients should involve inquiring from and educating them about the potential effects to serve as reinforcement.

### Conclusions

The findings show that majority of the patients have poor level of knowledge about oral anticoagulant therapy and knowledge about INR monitoring with warfarin therapy is highly substandard.

## Limitations

The study only included 207 participants and a larger sample size from different hospital settings is required to generalize the findings. Most patients were admitted in government hospitals, with little data obtained from private setups, and the majority of our sample population belonged to a low socioeconomic background and received little to no formal education, which could influence the results. There is a need for inclusion of moderate-high socioeconomic patients and patient with higher educational background in further studies in Pakistan. Second, the data was not divided according to the hospital it was collected from. This could have provided us with some insight into the effectiveness of patient counseling in the different setups. Furthermore, our questionnaire did not assess level of knowledge with duration of therapy or calculate a passing rate for the proportion of people with adequate knowledge.

## Supplementary information

**Additional file 1: Appendix S1.** Anticoagulant knowledge tool.

**Additional file 2: Table S1.** Knowledge scores.

## Data Availability

The datasets used and/or analyzed during the current study are available from the corresponding author on reasonable request.

## Abbreviations

OAC: Oral anti coagulant
INR: International normalised ratio
AKT: Anticoagulation knowledge tool
NSAIDs: Non-steroidal anti-inflammatory drugs
