# Supplementary material for: Are patients on oral anticoagulation therapy aware of its effects? A cross-sectional study from Karachi, Pakistan
Source: BMC Res Notes. 2020 Jun 9;13:279. doi: 10.1186/s13104-020-05119-w (PMC7285618; doi:10.1186/s13104-020-05119-w)
Supplement: Supplementary file 1 — Additional file 1: Appendix S1. Anticoagulant knowledge tool. [file 13104_2020_5119_MOESM1_ESM.docx]

**Appendix1: Anticoagulant Knowledge Tool**

**Introduction**

We thank you in advance for agreeing to fill in this questionnaire and we appreciate you taking the time to support this research. By completing this questionnaire, you will help in validating a tool that will be useful in caring for people taking anticoagulant medicines. Your responses, including demographic information will remain anonymous, and your confidentiality will be protected.

**Instructions on completing the questionnaire**

Please complete the following questions to reflect your opinions as accurately as possible and to the best of your knowledge.

- If you do not know the response to a question, please write ‘I don’t know’ in the space provided.
- If you are not sure of the response to a multiple choice question, please tick ‘not sure’ among the options provided.

Study Title: **Development and Validation of an Oral Anticoagulation Knowledge Tool (AKT)**

Link: *https://www.ncbi.nlm.nih.gov/pmc/articles/PMC4924828/*

This is a validated questionnaire to assess knowledge of oral anticoagulation in patients.

**Our study: Are Patients on Oral Anticoagulation Therapy Aware of its Effects? A Cross-sectional Study from Karachi, Pakistan**

**Questionnaire**

**Section 1: Demographic Information**

1. What is your gender?
2. Male
3. Female
4. How old are you? ……………….. years
5. What is the highest level of education you have completed?
6. High school or equivalent
7. College
8. Technical or vocational education
9. Bachelor’s degree
10. Postgraduate degree
11. No formal education
12. Occupation ..............................................................................................
13. Monthly family income USD...................................
14. How long have you been taking an oral anticoagulant medicine?
15. Less than 3 months
16. 3 -12 months
17. 1 -2 years
18. Greater than 2 years
19. I’m not taking an anticoagulant medication

**Section 2: Anticoagulation Knowledge**

**2.1 General questions**

1. What is the name of your anticoagulant medicine?

………………………………………………………………………………………………..

2. Why has your doctor prescribed you this medicine?

………………………………………………………………………………………………..

3. How does this medicine work in your body?

………………………………………………………………………………………………..

4. How many times a day do you need to take this medicine?

………………………………………………………………………………………………..

5. For how long do you need to take this medicine (for example, 3 months, and 6 months, life-long)?

………………………………………………………………………………………………..

6. Why is it important to take this medicine exactly as your doctor has told you?

………………………………………………………………………………………………..

7. Is it important to take this medicine at the same time each day?

a) Yes b) No c) Not sure

8. Is it okay to double the next dose of this medicine if you miss a dose?

a) Yes b) No c) Not sure

9. Is it possible that skipping one dose of this medicine could worsen your condition?

a) Yes b) No c) Not sure

10. Is it appropriate to stop taking this medicine once you feel better?

a) Yes b) No c) Not sure

11. Is it safe to take anti-inflammatory medicines like ibuprofen (Nurofen^®^ or Advil^®^) while you are taking this medicine?

a) Yes b) No c) Not sure

12. Is it safe to take vitamin supplements and herbal medicines with this medicine without consulting your doctor?

a) Yes b) No c) Not sure

13. Is there any benefit in taking more of this medicine than your doctor has told you to take?

a) Yes b) No c) Not sure

14. Will drinking too much alcohol increase the risk of side effects with this medicine?

a) Yes b) No c) Not sure

15. Would you inform a surgeon, dentist or other health professional that you are taking this medicine before undergoing surgery or a procedure?

a) Yes b) No c) Not sure

16. Is it important that all the health care practitioners you see know that you are taking this medicine?

a) Yes b) No c) Not sure

17. What is the most important side effect of this medicine?

………………………………………………………………………………………

18. THREE signs of side effects that you should watch out for while taking this medicine are:

………………………………………………………………………………………..

………………………………………………………………………………………..

………………………………………………………………………………………..

19. THREE things you can do to reduce your risk of side effects are:

………………………………………………………………………………………..

………………………………………………………………………………………..

………………………………………………………………………………………..

20. What is the best step to take if you accidentally take too much of this medicine?

………………………………………………………………………………………..

**2.2 Section 2**

1. What is your target INR range? ..................................

2. What was your last INR reading? …………...............

3. Are regular INR tests necessary to know how well this medicine is working?

a) Yes b) No c) Not sure

4. Is an INR value above your target range good for your general wellbeing?

a) Yes b) No c) Not sure

5. Is it possible for INR values below your target range to be bad for your health?

a) Yes b) No c) Not sure

6a. Is it possible for what you eat to affect your warfarin therapy?

a) Yes b) No c) Not sure

6b. If you answered ‘Yes’ above, list THREE foods that can affect your anticoagulant therapy.

……………………………………………………………………………………

……………………………………………………………………………………

……………………………………………………………………………………

7. List one vitamin that can significantly affect your anticoagulant therapy.

……………………………………………………………………………………
