# Supplementary material for: Are patients on oral anticoagulation therapy aware of its effects? A cross-sectional study from Karachi, Pakistan
Source: BMC Res Notes. 2020 Jun 9;13:279. doi: 10.1186/s13104-020-05119-w (PMC7285618; doi:10.1186/s13104-020-05119-w)
Supplement: Supplementary file 2 — Additional file 2: Table S1. Knowledge scores. [file 13104_2020_5119_MOESM2_ESM.docx]

**Table S1. Knowledge scores**

|  | **Anticoagulant Knowledge** | **INR knowledge** |
| --- | --- | --- |
| Mean Score±SD, % | 49.9±16.2 | 14.6±16.4 |
| Median Score (minimum-maximum), % | 48.7 (8.3-91.7) | 10.3 (0.0-70.0) |
| Number of people with >50% score | 86 | 4 |
| Percentage of people with >50% score | 41.5 | 1.9 |

INR, international normalized ratio; SD, standard deviation
